# Lumbosacral Transitional Vertebra Contributed to Lumbar Spine Degeneration: An MR Study of Clinical Patients

**DOI:** 10.3390/jcm11092339

**Published:** 2022-04-22

**Authors:** Linxiang Cheng, Chao Jiang, Jiawei Huang, Jiale Jin, Ming Guan, Yue Wang

**Affiliations:** Spine Lab, Department of Orthopedic Surgery, The First Affiliated Hospital, Zhejiang University School of Medicine, Hangzhou 310003, China; q773916513@zju.edu.cn (L.C.); gzc_1993@zju.edu.cn (C.J.); huangjw@shanghaitech.edu.cn (J.H.); 22018143@zju.edu.cn (J.J.)

**Keywords:** lumbosacral transitional vertebra (LSTV), magnetic resonance (MR), disc degeneration, Modic changes, endplate defects

## Abstract

We aimed to comprehensively characterize degenerative findings associated with various types of lumbosacral transitional vertebra (LSTV) on magnetic resonance images. Three hundred and fifty patients with LSTV (52.3 ± 10.9 years), including 182 Castellvi type I, 107 type II, 43 type III, and 18 type IV, and 179 controls without LSTV (50.6 ± 13.1 years), were studied. Discs, endplates, and posterior vertebral structures were assessed and compared to those of controls for the most caudal three discs on MRIs. There were no differences in degenerative findings between patients with type I LSTV and controls. For types III and IV, the transitional discs had smaller sizes, lower Pfirrmann scores, and lower rates of disc bulging (2.3% and 5.6% vs. 39.1%), osteophytes (2.3% vs. 15.1%), disc herniation (2.3% and 5.6% vs. 31.8%), and Modic changes (2.3% and 5.6% vs. 16.8%) than controls. However, the cranial discs had more severe Pfirrmann scores, disc narrowing and spinal canal narrowing, and greater rates of disc herniation (41.9% and 50.0% vs. 25.7%), endplate defects (27.9% and 33.3% vs. 14.4%) and spondylolisthesis (18.6% vs. 7.3%) than controls. Type II LSTV was associated with degenerative findings in the cranial segments but to a lesser degree, as compared with type III/IV LSTV. Thus, Castellvi type III/IV LSTV predisposed the adjacent spinal components to degeneration and protected the transitional discs. Type II LSTV had significant effects in promoting transitional and adjacent disc degeneration. Type I LSTV was not related to spinal degeneration.

## 1. Introduction

Lumbosacral transitional vertebra (LSTV) is a common anatomical variation at the lumbosacral junction of the spine [1,2], with an occurrence rate of 15.8–35.6% in general populations [3,4,5,6]. Although the etiology of LSTV remains unclear [7], it is generally accepted that LSTV is a congenital anomaly. There is a trend of family aggregation of LSTV [8], and the key genes in axial skeletal segmentation during embryogenesis, Hox-10 and Hox-11, have been implicated in the pathogenesis of LSTV [9,10]. In the presence of LSTV, the lumbar vertebral transverse processes may connect with the ala of sacrum to form a pseudoarticulation or bony fusion. On anteroposterior radiographs, Castellvi categorized LSTV into four subtypes: type I (transverse process > 19 mm), type II (pseudoarticulation between the lumbar vertebral transverse process and sacrum), type III (bony fusion between the transverse process and sacrum), and type IV (pseudoarticulation at one side and bony fusion at another). For types I–III, LSTV can be further classified as unilateral (a) or bilateral (b) [11].

LSTV may have important implications in spine practice. Other than vertebra numbering in radiological diagnosis and surgical planning, LSTV may have concomitant anatomical variations of the iliac artery [12]. More importantly, LSTV may be associated with back pain. As early as 1917, Bertolotti reported that back pain was more common in patients with LSTV than in those without [13]. While some population-based studies confirmed that LSTV was associated with the occurrence and severity of back pain and buttock pain [4,5], many others did not observe such associations [8,14,15]. Whether LSTV is a pathology related to back pain thus deserves further investigation.

Moreover, evidence suggests that LSTV may play a role in the development of degenerative lumbar disorders. While the disc cranial to the transitional segment may have more degeneration on magnetic resonance images (MRI) than other discs, the disc at the transitional segment tends to be mechanically protected and have fewer degenerative changes [16]. Another study reported that disc degeneration and facet degeneration in young men were most common in LSTV type I but less severe as compared with other types of LSTV [17]. It seems that LSTV may impact the proximal spinal segments, and such effects vary in degree among different types of LSTV. Yet, MR image features of LSTV have not been specifically depicted in detail.

Using clinical patients, the purpose of the current study is to characterize degenerative findings in the disc, endplate, spinal canal, and facet joints for various types of LSTV to understand the associations of LSTV with lumbar spine degeneration on MRIs.

## 2. Materials and Methods

### 2.1. Subjects

This is a cross-sectional study of clinical images. On the Picture Archiving and Communication Systems (PACS) at the author’s hospital, a general search was performed to identify patients who had consecutive computerized tomography (CT) and MR images of the lumbar spine between January 2016 and January 2018. The subject was excluded, if he or she had: (1) a previous history of lumbar spine surgery; (2) degenerative scoliosis or non-degenerative pathologies in the lumbar spine which could interrupt the evaluation of lumbar spine degeneration, including spinal tumors, infections, and traumatic fractures; (3) images with poor quality. Lumbar spine CT and MR images were then evaluated for included subjects.

### 2.2. Image Evaluation

MR imaging of the lumbar spine was performed with a 1.5 T scanner (GE, Boston, MA, USA), including sagittal T1-weighted (T1W), T2-weighted (T2W), and axial T2W sequences. CT was performed using a LightSpeed scanner (GE, Boston, MA, USA). Sagittal CT slices were then reconstructed, with a slice thickness of 3 mm.

**Figure 1:**
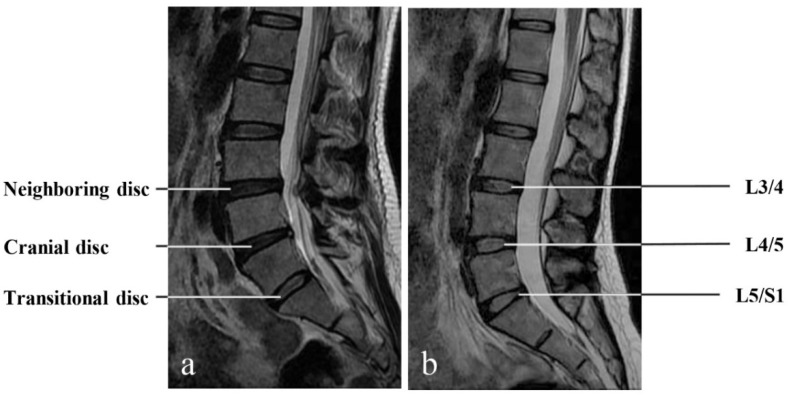
The most caudal three intervertebral discs were evaluated on T2W sagittal MR images. In the presence of LSTV, they were defined as the transitional disc, the cranial disc, and the neighboring disc (**a**), which corresponded to L5/S1, L4/5, and L3/4 in controls without LSTV, respectively (**b**). Spinal level-specific comparisons were performed for various measurements between LSTV patients and controls.

General image evaluation was performed on a computer station using the PACS system. The degree of disc degeneration was evaluated for the most caudal three intervertebral discs on T2W sagittal MRIs. In the presence of LSTV, it is sometimes difficult to number a lumbar vertebra, as there may be four or six lumbar vertebrae due to the lumbosacral anomalies. As such, we defined the disc at the transitional segment as the transitional disc, which is the most caudal and well-formed disc, the disc immediately cranial to the transitional segment as the cranial disc, and the disc proximately neighboring the cranial disc as the neighboring disc (Figure 1). Spinal-level-specific measurements of the transitional, cranial, and neighboring discs were compared to L5/S1, L4/5, and L3/4 in controls without LSTV, respectively.

### 2.3. LSTV Evaluation

Using Castellvi classification, two orthopedic surgeons (LC and MG, who had 4 years of expertise with spine MRIs) independently evaluated the presence or absence of LSTV on the reconstructed CT images, which is the gold standard for the evaluation of LSTV [6,18]. If present, LSTV was classified as type I-IV, and then further marked as “a” if unilateral, or “b” if bilateral [11]. Kappa statistics were used to examine the interobserver agreement for LSTV. When there was a disagreement between two evaluators, the case was resolved by discussion.

### 2.4. Disc Degeneration

On T2W sagittal MRIs, disc degeneration was evaluated using the Pfirrmann scale [19]. Individual phenotypes of disc degeneration were further assessed. Disc bulging, defined as the disc tissues exceeding the posterior edges of the vertebral bodies, was rated as absent or present [20,21]. Similarly, the absence or presence of osteophytes was assessed [22]. Disc narrowing was assessed as none, mild, moderate, or severe [23]. Using *ImageJ* (Version 1.80, NIH, Bethesda, MD, USA), the anterior, middle, and posterior disc heights were quantitatively measured and averaged for each disc [24]. In addition, the absence or presence of disc herniation, defined as the displacement of disc material beyond the normal margins of the disc space, was assessed [25].

Moreover, cerebrospinal-fluid (CSF)-adjusted disc signal intensity was measured on the mid-sagittal T2W MRIs using *ImageJ* [24]. In brief, the mean signal of the disc was acquired, and the signal of the CSF region behind the disc was obtained as a reference to adjust the signal of the corresponding disc [26]. Using *Spine Explorer* (Yitian), the sagittal area and anteroposterior diameter of the disc were automatically obtained on sagittal MRIs. The reliability and validity of this program in measuring MRIs have been reported elsewhere [24].

### 2.5. Endplate Degeneration

Modic changes and signal variations on the vertebral endplate and marrow were assessed on T1W and T2W sagittal MRIs. If present, Modic changes were further classified as type I, II, or III [27]. Endplate defects were defined as the loss or disruption of the smooth appearance of the endplate [28]. If present, endplate defects were further classified as focal, corner, or erosive defects, as previously reported.

### 2.6. Canal Stenosis, Ligamentum Flavum Thickening, and Facet Joint Degeneration

On T2W axial MRIs, spinal canal narrowing was assessed and further classified as normal, mild to moderate, or severe, as previously defined [29]. The thickness of ligamentum flavum was measured using *ImageJ*, and was rated as present if ≥4 mm on either side [25]. Using Weishaupt’s approach, facet joint degeneration was rated as none, normal, mild to moderate, or severe [30]. In addition, spondylolisthesis was evaluated as absent or present [25].

### 2.7. Statistical Analysis

Descriptive statistics were used to depict MR findings for various types of LSTV and controls. Measurements of the transitional disc, cranial disc, and neighboring disc in LSTV patients were compared to those of L5/S1, L4/5, and L3/4 discs in controls, respectively. Differences in MR findings among LSTV subtypes and controls were compared. A t-test was used for age and quantitative variables. Chi-square tests were used for measurements of disc bulging, osteophytes, disc herniation, Modic changes, endplate defects, and spondylolisthesis. Mann-Whitney U tests were used for ordinal variables, including Pfirrmann score, disc narrowing, spinal canal stenosis, ligamentum flavum thickening, and facet joint degeneration. The associations of age with Pfirrmann score, disc height, disc signal, and age were determined using linear regression. Statistical analysis was performed using *Stata* (Version 13.1, StataCorp LP, College Station, TX, USA). A *p*-value < 0.05 was considered statistically significant.

## 3. Results

### 3.1. Subject Characteristics

During the defined period, a total of 635 patients with consecutive CT and MR images of the lumbar spine were identified. Among them, 106 patients were excluded (25 had a history of spine surgery, 29 had lumbar vertebral fractures, 15 had spinal infections, 9 had scoliosis, 12 had lumbar tumors, and 16 had images of poor quality). As a result, 529 patients (238 men and 291 women, age 51.7 ± 11.7 years) were studied. Among them, 350 (66.2%) patients had LSTV, including 182 (34.4%) type I, 107 (20.2%) type II, 43 (8.1%) type III, and 18 (3.4%) type IV. The other 179 patients without LSTV were included as controls. 

There was no statistical difference in age between LSTV patients and controls (*p* = 0.12). The prevalence of LSTV was higher in men than in women (70.5% vs. 51.0%, *p* < 0.001). However, the prevalence rate of each LSTV subtype was not different between men and women (*p* > 0.05 for all). No statistical difference in degenerative findings were observed between men and women (*p* > 0.05 for all). For LSTV evaluation, the interobserver Kappa was 0.83 and was considered excellent.

### 3.2. Differences in MRI Findings between Unilateral and Bilateral LSTV

For LSTV types I, II, and III, we compared unilateral (a) and bilateral (b) MR findings, and no statistical differences in degenerative findings were observed between unilateral and bilateral LSTV (*p* > 0.05 for all). As such, data of unilateral and bilateral LSTV were merged for further analysis.

### 3.3. MRI Findings for Type I LSTV

**Figure 2:**
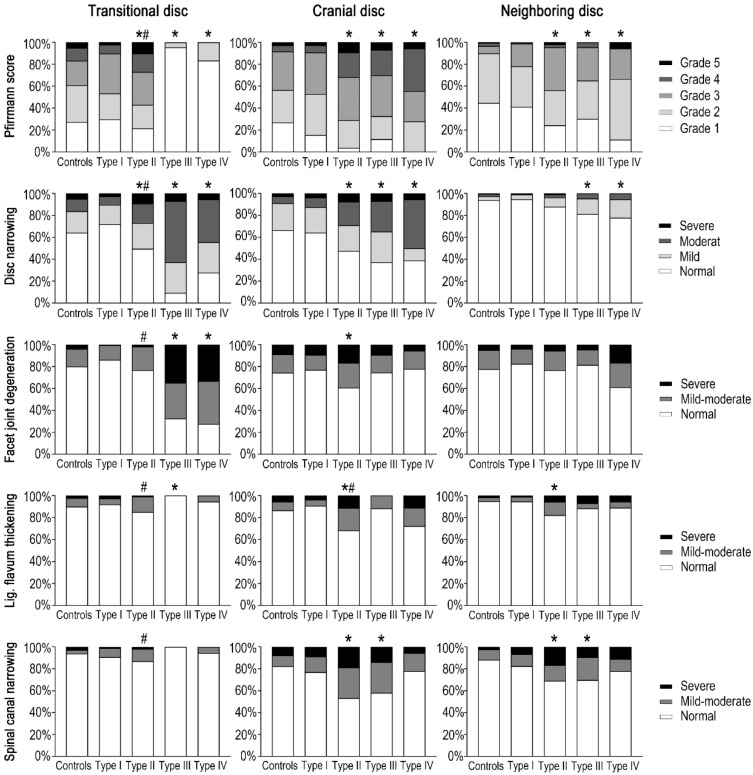
Pfirrmann scores, disc narrowing, facet joint degeneration, ligamentum flavum thickening, and spinal canal narrowing among patients with LSTV type I (N = 182), type II (N = 107), type III (N = 43), type IV (N = 18), and controls (N = 179). *: *p* < 0.05; measurements of the controls were the references. #: *p* < 0.05; measurements of type II were the references.

Between patients with type I LSTV and controls, there were no statistically significant differences in any degenerative findings for the most caudal three lumbar discs (*p* > 0.05 for all, Table 1 and Figure 2).

### 3.4. MRI Findings for Type II LSTV

(1) The Transitional Disc

Compared with the L5/S1 discs in controls, the transitional discs in type II LSTV had significantly greater rates of disc bulging (51.4% vs. 39.1%, *p* = 0.043), osteophytes (28.0% vs. 15.1%, *p* = 0.008), and endplate defects (22.4% vs. 12.2%, *p* = 0.034), and greater Pfirrmann scores and disc narrowing (Table 1 and Figure 2). Greater age was associated with higher Pfirrmann scores, lower disc heights, and lower disc signals of the transitional discs in type II LSTV, similar to the L5/S1 discs in controls, as well (*p* < 0.05, Table 2).

(2) The cranial disc

The cranial discs in type II LSTV patients had significantly higher rates of disc bulging (87.9% vs. 53.6%, *p* < 0.001), osteophytes (39.3% vs. 22.9%, *p* = 0.003), disc herniation (40.2% vs. 25.7%, *p* = 0.010), Modic changes (22.4% vs. 12.8%, *p* = 0.034), endplate defects (35.5% vs. 14.4%, *p* < 0.001), and spondylolisthesis (15.0% vs. 7.3%, *p* = 0.037), and had greater Pfirrmann scores, disc narrowing, facet joint degeneration, ligamentum flavum thickening, and spinal canal narrowing, as compared with the L4/5 discs in controls (Table 1 and Figure 2).

(3) The neighboring disc

The neighboring discs in type II LSTV patients had higher rates of disc bulging (54.2% vs. 24.0%, *p* < 0.001), osteophytes (39.3% vs. 27.4%, *p* = 0.037), disc herniation (13.1% vs. 4.5%, *p* = 0.008), endplate defects (20.6% vs. 7.5%, *p* = 0.003), and greater Pfirrmann scores, ligamentum flavum thickening, and spinal canal narrowing, as compared with the L3/4 discs in controls (Table 1 and Figure 2).

### 3.5. MRI Findings for Type III LSTV

(1) The Transitional Disc

The transitional discs in type III LSTV had significantly less anteroposterior diameters, lower disc heights, less area, and higher disc signals on the mid-sagittal MRI than the control L5/S1 discs (*p* < 0.05 for all, data not presented). Age was not associated with Pfirrmann score, disc height, or disc signal measurement of the transitional discs (*p* > 0.05, Table 3).

The transitional discs in type III LSTV had significantly lower rates of disc bulging (2.3% vs. 39.1%, *p* < 0.001), osteophytes (2.3% vs. 15.1%, *p* = 0.021), disc herniation (2.3% vs. 31.8%, *p* < 0.001), Modic changes (2.3% vs 16.8%, *p* = 0.013), and had lower Pfirrmann scores and ligamentum flavum thickening, but more severe disc narrowing and facet joint degeneration than the L5/S1 discs in controls (Table 1 and Figure 2). When compared to those in type II LSTV, the transitional discs in type III LSTV also had fewer degenerative findings in the discs, endplates, and facet joints (*p* < 0.05 for all, Table 1 and Figure 2).

(2) The cranial disc

The cranial discs in type III LSTV had significantly higher rates of disc herniation (41.9% vs. 25.7%, *p* = 0.036), endplate defects (27.9% vs. 14.4%, *p* = 0.028) and spondylolisthesis (18.6% vs. 7.3%, *p* = 0.022), and had greater Pfirrmann scores, disc narrowing, and spinal canal narrowing than the L4/5 discs in the controls (Table 1 and Figure 2).

(3) The neighboring disc

The neighboring discs in type III LSTV had higher rates of disc bulging (51.2% vs. 24.0%, *p* < 0.001) and spondylolisthesis (9.3% vs. 1.1%, *p* = 0.014), and higher Pfirrmann scores, more severe disc narrowing, and greater spinal canal narrowing than the L3/4 discs in controls (Table 1 and Figure 2).

### 3.6. MRI Findings for Type IV LSTV 

Similar to type III LSTV, in type IV LSTV, degenerative findings were barely observed in the transitional disc but rather common in the cranial disc (Table 1 and Figure 2). For the most caudal three discs, no significant differences in degenerative findings were observed between type IV and type III LSTV (*p* > 0.05 for all). In addition, the neighboring discs in type IV LSTV had higher rates of disc bulging (50.0 % vs. 24.0%, *p* = 0.017), endplate defects (33.3% vs. 7.5%, *p* = 0.001), and spondylolisthesis (11.1% vs. 1.1 %, *p* = 0.042) than the L3/4 discs in controls.

## 4. Discussion

In this study, MRI features of each LSTV type were characterized to understand their associations with lumbar spine degeneration. MR findings in type I LSTV spines were not different from those of age-matched controls, suggesting that type I LSTV did not have specific degenerative effects on the lower lumbar spine. Similarly, type III and IV LSTV were comparable in the context of lumbar spine degeneration. Compared with the L5/S1 discs in controls, the transitional discs in type III and type IV LSTV were smaller in size and degenerative findings were rare. On the contrary, the disc cranial to the transitional segment had serious degenerative changes in the disc and endplate, suggesting that type III and IV LSTV were associated with adjacent disc and endplate degeneration. On the other hand, type II LSTV was also related to cranial disc degeneration.

LSTV has important clinical implications in spine-related practice. Reportedly, over 30% of adults in general population have lumbosacral transition, and LSTV has long been suspected as a cause of common back pain [13]. On the other hand, it is sometimes challenging to number a lumbar vertebra in the presence of LSTV [31], resulting in accidental confusion in clinical communication and surgery. A less studied area of LSTV, however, is that LSTV may play an important role in lumbar spine degeneration and related disorders.

Recently, spinal sagittal balance and spinopelvic alignment have become important evaluations in the research and clinical practice of degenerative lumbar spinal disorders [32,33,34]. Although the Roussouly classification of sagittal spinal profile is widely used, its association with various subtypes of LSTV remains understudied [35,36]. Since LSTV has significant influence on pelvic morphology and spinopelvic parameters, reliable identification of LSTV and proper selection of measurement points are of great clinical relevance. For example, Henryk reported that LSTV was associated with changes in fixed spinopelvic parameters, including pelvic radius, pelvic incidence, and sacral table angle [37]. Nevertheless, the associations of LSTV with sagittal alignment and lumbar spinal degenerative findings deserve further study.

In type III and IV transitions, the discs at the transitional segments had much smaller sizes but higher signals than other lumbar discs and the control L5/S1 discs. The absence of associations between age and Pfirrmann score and disc signal of the transitional discs in type III/IV LSTV suggest that the transitional discs were small in size at birth and further escaped from age-related degeneration. Such phenomena may be attributable to the mechanical shielding effect of osseous fusion in the transitional segment. Further, degenerative findings were common in the cranial segment in both type III and IV LSTV. Together, findings suggest that type III/IV LSTV may have profound effects of promoting degeneration on the cranial segment. Related mechanisms may involve abnormal torque moments [11], altered mechanical stress [38], transferred mobility on the cranial segment [39], and weak iliolumbar ligament at the adjacent segment [40]. Notably, in this study, 41.9% of type III and 50% of type IV LSTV at the cranial discs had radiological disc herniation (Table 2), suggesting that type III/IV may be an important risk factor for lumbar disc herniation. In fact, cranial disc degeneration in type III/IV LSTV is similar to the adjacent segment disease following spinal instrumentation and fusion [41,42].

In this study, type II LSTV, with one or two enlarged L5 vertebral processes articulating with the sacrum, were associated with disc degeneration at both the cranial and transitional segments. Contrary to a previous study which reported that type II LSTV protected the transitional discs from degeneration [16], we observed a greater degree of degeneration at the transitional discs in type II LSTV than in the controls. Although some studies suggested that LSTV are genetically formed [43,44], we noticed that age was associated with disc degeneration in the transitional segment. It is possible that type II LSTV is an acquired lumbosacral anomaly formed as a result of progressive disc height decrease in aging. Further evidence is needed to support this new hypothesis.

Although it is traditionally counted as type I LSTV when the transverse processes of L5 vertebra are relatively large (>19 mm), such morphological enlargement was not related to lumbar spine degeneration [11]. Consistent with many studies [11,45], no differences in degeneration measurements were observed between type I LSTV and controls in this study. In addition, there was no anatomical connection between the vertebral transverse process and sacrum. Our findings suggested that Castellvi type I may not be a true lumbosacral anomaly and could be excluded from LSTV [40,45,46].

This was merely a retrospective image study of clinical patients. Detailed protocols were used to characterize various degenerative findings of multiple spinal components in the lower lumbar spine for each type of LSTV. Furthermore, LSTV was classified on CT images, which was more accurate than the traditional anteroposterior radiograph [18]. While the study samples were selected based on consecutive patients, most were with back or leg pain. Using highly selected clinical patients, this image study focused on MR findings of LSTV, and epidemiological parameters derived for LSTV should not be directly compared to those from general populations. In addition, the associations of LSTV with clinical symptoms were not studied, which was an obvious limitation. 

In summary, types II-IV LSTV, particularly types III and IV, were closely associated with lumbar spinal degeneration. Type I may not be a true lumbosacral anomaly.

## 5. Conclusions

Castellvi types III and IV LSTV predisposed the adjacent spinal components to degeneration and protected the transitional discs from age-related degeneration. Type II LSTV had significant effects in promoting transitional and adjacent disc degeneration. Type I LSTV was not related to lumbar spine degeneration.

## Figures and Tables

**Table 1:** Degenerative findings on lumbar MRIs for patients with LSTV and controls.

	Controls (N = 179)	Type I (N = 182)	Type II (N = 107)	Type III (N = 43)	Type IV (N = 18)
**Disc bulging**					
Neighboring disc	43 (24.0%)	59 (32.4%)	58 (54.2%) *	22 (51.2%) *	9 (50.0%) *
Cranial disc	96 (53.6%)	111 (61.0%)	94 (87.9%) *	**24 (55.8%) ^#^**	12 (66.7%)
Transitional disc	70 (39.1%)	78 (42.9%)	55 (51.4%) *	1 (2.3%) *^#^	1 (5.6%) *
**Osteophytes**					
Neighboring disc	49 (27.4%)	36 (19.8%)	42 (39.3%) *	10 (23.3%)	5 (27.8%)
Cranial disc	41 (22.9%)	36 (19.8%)	42 (39.3%) *	15 (34.9%)	9 (50.0%) *
Transitional disc	27 (15.1%)	22 (12.1%)	30 (28.0%) *	1 (2.3%) *^#^	1 (5.6%)
**Disc herniation**					
Neighboring disc	8 (4.5%)	11 (6.0%)	14 (13.1%) *	5 (11.6%)	2 (11.1%)
Cranial disc	46 (25.7%)	46 (25.3%)	43 (40.2%) *	18 (41.9%) *	9 (50.0%) *
Transitional disc	57 (31.8%)	42 (23.1%)	27 (25.2%)	1 (2.3%) *^#^	1 (5.6%) *
**Modic changes**					
Neighboring disc	13 (7.3%)	15 (8.2%)	12 (11.2%)	2 (4.7%)	2 (11.1%)
Cranial disc	23 (12.8%)	29 (15.9%)	24 (22.4%) *	8 (18.6%)	5 (27.8%)
Transitional disc	30 (16.8%)	35 (19.2%)	20 (18.7%)	1 (2.3%) *^#^	1 (5.6%)
**Endplate defects**					
Neighboring disc	27 (7.5%)	12 (6.6%)	22 (20.6%) *	6 (14.0%)	6 (33.3%) *
Cranial disc	52 (14.4%)	27 (14.8%)	38 (35.5%) *	12 (27.9%) *	6 (33.3%) *
Transitional disc	44 (12.2%)	21 (11.5%)	24 (22.4%) *	**2 (4.7%) ^#^**	1 (5.6%)
**Spondylolisthesis**					
Neighboring disc	2 (1.1%)	3 (1.6%)	4 (3.7%)	4 (9.3%) *	2 (11.1%) *
Cranial disc	13 (7.3%)	12 (6.6%)	16 (15.0%) *	8 (18.6%) *	1 (5.6%)
Transitional disc	7 (3.9%)	3 (1.6%)	2 (1.9%)	0 (0%)	0 (0%)

*: *p* < 0.05; measurements of the controls were the references. ^#^: *p* < 0.05; measurements of type II were the references. Data with statistical differences were displayed in bold.

**Table 2:** Linear regression analysis of various parameters of transitional discs with age in type II LSTV.

	Coefficient	95% CI	*p* Value
**Pfirrmann score**	0.027	(0.001, 0.054)	0.044 *
**Disc height**	−0.108	(−0.186, −0.030)	0.007 *
**Disc signal**	−0.003	(−0.004, −0.001)	0.007 *

*: *p* < 0.05; measurements of the controls were the references. CI: Confidence interval. Data with statistical differences were displayed in bold.

**Table 3:** Linear regression analysis of various parameters of transitional discs with age in type III LSTV.

	Coefficient	95% CI	*p* Value
**Pfirrmann score**	0.002	(−0.003, 0.007)	0.436
**Disc height**	0.009	(−0.035, −0.055)	0.666
**Disc signal**	0.001	(−0.003, 0.004)	0.788

Measurements of the controls were the references. CI: Confidence interval. Data with statistical differences were displayed in bold.

## Data Availability

The data presented in this study are available on request from the corresponding author. The data are not publicly available due to privacy restrictions.
